# A feminist political ecology of agricultural innovations in smallholder farming systems: Experiences from wheat production in Morocco and Uzbekistan

**DOI:** 10.1016/j.geoforum.2023.103865

**Published:** 2023-11

**Authors:** Dina Najjar, Hanson Nyantakyi-Frimpong, Rachana Devkota, Abderrahim Bentaibi

**Affiliations:** a Sustainable Intensification and Resilient Production Systems Program (SIRPSP), The International Center for Agricultural Research in the Dry Areas (ICARDA), Rabat, Morocco; b Department of Geography and the Environment, University of Denver, CO, USA; c School of Environmental Design and Rural Development, University of Guelph, Canada; d Institut National de la Recherche Agronomique (INRA), Meknes, Morocco

**Keywords:** Innovations, Wheat, Feminist political ecology, Technology adoption

## Abstract

A clear consensus has emerged that innovations are important for adapting to drought and overcoming other biophysical limitations in smallholder farming systems; however, women are notably marginalized from agricultural innovations. We examine whether and how gendered roles and responsibilities shape the adoption and usage of improved wheat varieties and simultaneously uncover opportunities to address and lessen gender-based differences in agricultural innovations. The field data were collected using snowball sampling from seven communities (three in Morocco and four in Uzbekistan) among 574 farmers (half men and half women) of different generations, genders, social statuses, and social classes. Our findings demonstrate how the complex interactions of biophysical constraints, intra-household (spousal and kinship) relations, and the broader macro-level political economy of agriculture converge to influence different identities of women and men farmers’ wheat production and processing practices. We argue that without focusing on the socio-cultural factors affecting agriculture, new seed varieties alone cannot address the multifaceted problems confronting farmers in all parts of the world.

## 1 Introduction

The socio-ecological drivers of agricultural innovations have been a major focus of study in the human-environment sciences for at least three decades (e.g., Borremans et al., 2018, Doss and Morris, 2000, Louah et al., 2017, Weyori et al., 2018). Over these years, a clear consensus has emerged that innovations are important for adapting to environmental change and overcoming other biophysical limitations in smallholder farming systems (Borremans et al., 2018, Eastwood et al., 2017, Weyori et al., 2018). This body of work has also shown that women are often largely marginalized from agricultural innovations (Peterman et al., 2010, Seymour et al., 2016), although they are heavily engaged in food production (Doss & Morris, 2000). Women, in particular those of less resource endowment and women who are heads of households, are likely to fare worse in adopting innovations (Beuchelt, 2016). Our understanding of innovations is informed by Berdegue’s (2005, p. 3) definition of innovation as “social constructs, and as such, they reflect and result from the interplay of different actors, often with conflicting interests and objectives, and certainly with different degrees of economic, social, and political power.”.

Many studies also suggest that women’s burden increases with the introduction of new agricultural technologies (e.g., Gebre et al., 2019, Halbrendt et al., 2014). In some contexts, introducing new technologies affects labor, land, and resource allocation patterns between men and women (Doss 2001). Given women’s roles in agriculture, food security, nutrition, and family well-being (Quisumbing et al., 2014), it is important to understand how their innovation capacity could be strengthened. Although a fair amount of attention has been paid to the determinants of technology adoption in the economic development literature (e.g., Doss and Morris, 2000, Quisumbing et al., 2014), much less attention has been given to understanding both the role of intersectionality and the gender-specific constraints to agricultural innovations that include agronomic and resources management practices.

The primary objective of this paper is to examine whether and how gendered roles and responsibilities shape the adoption and usage of improved wheat varieties (Theis et al., 2018). A secondary objective is to uncover opportunities to address and lessen gender-based differences in agricultural innovations. Using a feminist political ecology lens (Elmhirst, 2015, Vaz-Jones, 2018, Vercillo, 2021), we pay attention to how micro-level gender politics intersect with biophysical constraints and macroeconomic dynamics to shape wheat innovations. We specifically ask two questions:

How do gender labuor roles, relations, and responsibilities inform decisions about adopting improved wheat varieties among smallholder farmers?
How do women and men farmers (of different generations, social classes, and social statuses in different locations) perceive biophysical constraints and broader macroeconomic factors in adopting wheat innovations?

These questions are important because they go directly to the issue of whether gender-related differences in the adoption of agricultural innovations could be attributed to inherent characteristics of improved technologies themselves or result from other external factors. The empirical material for the paper comes from comparative, in-depth household-level data from Morocco and Uzbekistan.

To answer the above research questions, we first present our theoretical approach, which draws from feminist political ecology, and then describe the two research countries. Next, we describe our methodology before presenting the research findings, organized into three key parts related to feminist political ecology. The first part focuses on biophysical constraints that hinder wheat innovations in Morocco and Uzbekistan. The second part reveals how different forms of micro-level gender politics and intersectional differences come together to shape the adoption and benefits of wheat innovations. In the third and final sub-section of the analysis, we look at how broader macro-scale politics also influence agricultural innovations. The concluding section discusses how to support well-targeted and socially inclusive innovations in smallholder agriculture. We also discuss how this paper advances empirical and theoretical work in feminist political ecology.

## 2 Conceptual framework: Gender norms, agency, and agricultural innovations

Innovations are important for adapting to drought and overcoming other biophysical limitations in agricultural production systems. However, critical analyses of the inequities associated with the first Green Revolution, and how these may be salient in the contemporary second green/gene revolution unfolding in Africa and South Asia has shown that innovations are not always good for local ecosystems, nor do they always foster positive social change (Feldman and Biggs, 2012, Kerr, 2012, Patel, 2013). More recent research has shown that climate smart innovations and high yielding varieties sponsored by donors, development agencies and private–public partnerships have led to exacerbating social and gender inequalities, reduced resilience to climate change while benefitting input companies, and these studies call for innovations that are more adapted to varying and local needs of women and men farmers (Moseley et al., 2015, Clay and Zimmerer, 2020).

Adoption of innovations is also a social process largely impacted by gender norms, or what men and women are allowed or not allowed to do (Badstue et al., 2017, Cohen et al., 2016, Kingiri, 2013). Case studies from different geographical and cultural settings (e.g., Ethiopia, India, Nepal, Bangladesh, and Morocco) suggest that compared to women, men are more able to adopt new wheat varieties (e.g., Badstue et al., 2017). This is also partially due to external contexts, which better enable men’s access to financial resources and institutional interactions (Badstue et al., 2017, Farnworth et al., 2018). Based on past gender and innovation studies in India, Ethiopia and other settings, women are often reported to be largely marginalized from innovations (e.g., see Badstue et al., 2017). As such, it is important to understand how their innovation capacities can be strengthened. To gain insights into women’s agency for innovations, it is important to go beyond simple comparisons between women and men and consider which men and women are adopting agricultural innovations, in which biophysical, external, and local contexts, as well as why.

People are subjected by often competing but also colluding forms of social difference. Thus, our task becomes to explore the exercise of power and how forms of perceived social differences such as gender, age, kinship, and class relations are enrolled at various dimensions of society (Nightingale, 2011, Nyantakyi-Frimpong, 2020). Equally important is to understand how external political and economic dynamics play out on the ground. Policy is especially important in agriculture as it affects crop choices, the inputs used, seeds available for farmers to grow, and land tenure. Drawing from such insights, this paper will apply a nuanced understanding of gender as a social construction of identities over time and space (Elmhirst, 2015). We explore how gender intersects with other identities and how these are affected by external policies and biophysical dynamics to shape the uptake of agricultural innovations and situated agency in dealing with critical challenges confronting rural households (Kansanga et al., 2019).

In this paper, the assessment of how gender intersects with other forms of difference is motivated by the work of Crenshaw (1990), who first coined the term *intersectionality* to describe how race and gender bias can converge to produce social injustices (see also Crenshaw, 1989). Drawing largely from insights from Crenshaw (1990), there is now a growing body of work on intersectional feminist political ecology (Cole, 2017, Senanayake, 2022), most of which have focused specifically on agriculture-related issues (e.g., Kansanga et al., 2019, Lawson et al., 2020, Nyantakyi-Frimpong, 2017, Nyantakyi-Frimpong, 2020, Vercillo, 2021).

Following earlier work in feminist political ecology (e.g., Carney and Watts, 1991, Schroeder, 1999), we argue that agricultural innovations and related knowledge are situated in material and gendered practices. Efforts to achieve lasting and equitable improvements of farm outcomes need to pay attention to processes and the intersectionality of gender, class, and other subjectivities at different scales that can access particular farming practices and knowledge in a given place (Kansanga et al., 2019, Vercillo, 2021). This paper, thus, draws attention to the relational emergence of space and society, challenging ideas of difference that rely on fixed subjectivities and emphasizing the importance of the everyday in the production of social and especially gender inequalities (Elmhirst, 2015, Nightingale, 2011). We will make the case that intersectional identities and their attendant roles and responsibilities, which in turn are determined by gender norms, as well as biophysical and institutional context, shape how households can adopt and adapt technological innovations.

Aside from intersectional analysis, work under feminist political ecology also seriously considers geographical scale. While the household and intrahousehold relations remain a key unit of analysis in understanding gender dynamics in much feminist political ecology research, there is equal attention given to macro-level factors such as the effect of government policies (Ahmed and Gasparatos, 2021, Kansanga et al., 2019, Vercillo, 2021). This form of scaler analysis is applied to the case study being examined in the current paper. Before presenting the case study findings, we first describe the research methodology.

## 3 Methodology

**Fig. 1:**
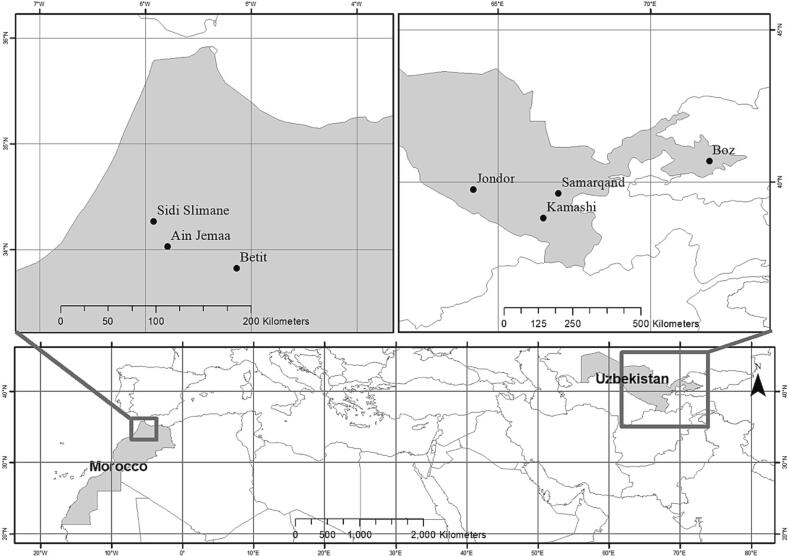
Map showing research sites in Morocco and Uzbekistan.

The field data were collected from seven communities (three in Morocco and four in Uzbekistan) among 574 farmers (82 participants (41 men and 41 women) in each of the seven communities) (See Table 1). Snowball sampling was used to select the respondents. The data for this study is part of GENNOVATE, a global research project whose aim was to understand linkages among gender norms, agency, and capacities to innovate (Feldman and Badstue, 2018). Interactions among gender, age, and social class were an explicit focus of this project (Petesch et al., 2018). Between 2014 and 2016 a series of case studies were conducted in four communities within Uzbekistan and three communities in Morocco as part of the GENNOVATE project. Four districts in Uzbekistan, which exemplify the four sample communities, were selected based on prior survey results from 1,400 households that addressed gendered decision-making power in managing household farms (Yigezu 2012). Based on this survey, conducted in 2012 in eight provinces, we selected four communities with the aim of maximizing diversity in decision-making power dynamics. The selection of study communities was partially based on prior relationships with researchers in the region. For instance, in Uzbekistan, we had prior local contacts with the Ministry of Agriculture and Water Resources (MoAWR) within our sample communities—Jondor in Bukhara, Kamashi in Kashkadarya, Pazdargom in Samarkand and Boz in Andijan (see Fig. 1). These prior relations contributed to easing access to respondents who met the criteria of this study.

**Table 1:** Data collection details adopted for this study in the two countries and seven communities.

Data Collection Methods	Study sites (total no. of activities*)Morocco (3) Uzbekistan (4)	Sample size per activity	Total Sample size 574
Focus group discussions (FGDs) with the middle class women and men farmers	6 (3 m, 3w) 8 (4 m, 4w)	10 participants in each FGD	140 (70 m, 70w)
FGDs with the low income women and men farmers	6 (3 m, 3w) 8 (4 m, 4w)	10 participants in each FGD	140 (70 m, 70w)
FGDs with older adolescents and young male and women adults	6 (3 m, 3w) 8 (4 m, 4 w)	10 participants in each FGD	140 (70 m, 70w)
Semi-structured interview with local innovator for innovator pathway	12 (6 m, 6w) 16 (8 m,8w)	2 men and 2 women	28 (14 m, 14w)
Interviews with individuals for life histories	12 (6 m, 6w) 16 (8 m,8w)	2 men and 2 women	28 (14 m, 14w)
Wage workers	36 (18 m, 18w) 48 (24 m, 24w)	6 men and 6 women	84 (42 m, 42w)
Community Leaders (Key informants)	6 (3 m,3w) 8 (4 m, 4 w)	1 man and 1 woman	14 (7 m, 7w)
Total	16 FGDs 24 FGDs 66 Interviews 88 Interviews	82 participants per site	574 participants

*All activities were conducted with half men and half women respondents.

This study included three areas of the Saiss region in Morocco: Ain Jemaa, Betit, and Sidi Slimane (see Fig. 1). These areas differ in terms of natural resource endowments and labor markets. Ain Jemaa is rain-fed, typically grows subsistence food crops such as wheat, chickpeas, fava beans, and olives. Most agricultural work is carried out by family labor. Demand for paid agricultural labor is relatively low but often a little higher during planting, weeding, and harvesting seasons. The other two areas, namely, Betit and Sidi Slimane, have received extensive irrigation infrastructure in recent years, owing to the Green Morocco Plan (GMP), making more commercial cultivation feasible. The GMP offers grants to cultivators for drip irrigation and wells at highly subsidized rates (Bossenbroek and Zwarteveen, 2015, Faysse, 2015). The premise is that sustainable intensification of agriculture and subsequent increases in yield are significantly enabled by irrigation access (Badraoui, 2014). The GMP, spanning from 2008 until 2020, offered microcredit to farmers’ collectives in an effort to address the dual objective of integrating farmers better into the market and increasing productivity (Faysse, 2015). The GMP was preceded by important agrarian reforms the impacts of which continue to unfold in present times. In 2008, for similar reasons the government of Morocco privatized land ownership from the soviet-inspired collective form of ownership in the Saiss region (Bossenbroek and Zwarteveen, 2015). This policy is part of ongoing efforts initiated in the 1980s toward liberalizing the economy, favouring productivity and competitive agricultural sector over addressing social inequalities (Jouve, 2002). Because only one person, often a man, can own the land, the privatization of land has fuelled landlessness and family feuds, marginalizing men and especially women from owning land (Bossenbroek and Zwarteveen, 2015). In Morocco, the cases were selected based on project interventions related to various agronomic practices on different crops, chickpeas, fava beans, and wheat.^5^

In this study, we conducted 16 focus group discussions (FGDs) in Morocco and 24 FGDs in Uzbekistan, half of which were with men and half with women, with ten participants in each group. The details on data collection methods and respondents are given in Table 1. The groups included a women’s and a men’s FGD from each of the middle and lower income classes, and two youth groups, similarly separating girls and boys in order to enhance the likelihood that people discuss their concerns freely. In each site, local leaders were asked to recruit participants in the various income groups based on local realities (e.g., land endowment, livestock ownership, educational attainment, car ownership, etc.). Youth selection criteria were that participants be between 16 and 24 years old. All FGD selection criteria included at least six participants who were actively engaged in agriculture and represented diverse marital statuses and families. In addition to the FGDs, 28 interviews were conducted with local innovators (two men and two women in each community), as were 28 life histories from men and women who were viewed as having improved their economic standing to aid in our understanding of what they defined as the conditions that led to either their success or failure to adopt wheat innovations. Life histories are aimed at understanding changes in relationships and economic standing through agriculture, labour, and accumulation or sales of assets throughout a life course.

Sensitive issues, including household ownership and control over resources, were discussed in one-on-one interviews. Key informant interviews were also held with a man and woman leader in each community to provide a window on general community characteristics, including women’s participation in public life, average size of land holding, and the development programs that were available for women and men in the community. In addition to this GENNOVATE data, twelve interviews were held with workers in each of the seven communities, six women and six men, that discussed the specific paid roles and the problems workers faced in agriculture, as well as their sources of agricultural, off-farm, and non-farm income. Innovation in this study was defined to respondents expansively to encompass agricultural technologies, natural resource management practices, learning opportunities, relationships, and institutions which are new for the study communities sampled. These innovations may be locally devised or externally introduced. Some of these innovations mentioned by the communities included new lending mechanisms/credit, new wheat varieties, new relations with research organizations, machinery, and irrigation mechanisms.

The two countries have similarities and differences in their institutional, technological, and gender norms dynamics. In both countries, religion of Islam figures out prominently in everyday practices and policy dynamics. Also, in both countries, outmigration of men leads to the feminization of agriculture and wheat is a main crop and staple food. Some of the major common trends in both countries have been highlighted in Table 2. Wheat is the single most important crop in both countries. By looking at similar agricultural innovations (varieties), biophysical constraints (climate-related), and lending mechanisms (credit), the role of contextual factors related to agency, policy, and intersectionality becomes more evident. In Uzbekistan we were able to interview both women who were managing farmland and women who were involved in the processing of wheat. Very few women were able to access land for commercial wheat production. We interviewed both types of women’s engagement with wheat innovations in our study. In Morocco, we were unable to find women who manage land; we interviewed women who support their husbands in farming the land as well as women involved in processing. From the wheat value chain perspective, in both countries women were far more likely to be involved in processing wheat by-products.

**Table 2:** Common trends in Morocco and Uzbekistan.

Trends in Morocco	Trends in Uzbekistan
Visibility issues with serious implications on women’s agency and wellbeing	Out-migration of men
Privatization of land	Re-collectivization
Change in irrigation	Land is a burden not an asset
Differences in traits and wheat innovation preferences	Differences in trait and wheat innovation preferences
Class-based and gender-based opportunity structure	Class-based and gender-based opportunity structure
Interlocking rights over land use (temporary and invisible roles for women)	Interlocking rights over land use (renter-owner agreements, ‘dakhan’ (smallholder) farmers mostly women, vegetables and livestock are women’s enterprises), farming land owned mostly by men
Women’s empowerment and gendered impacts of development projects are mixed as poor women can now find wage work but the working conditions are indecent (see Najjar et al. 2018)	Development programs’ gender-related effects are invisiblized and should be evaluated using intersectionality and a relational approach (see Kim et al., 2018)

Data were translated from local languages (Uzbek and Arabic) to English and coded with NVivo 10 for major themes, namely mentions of biophysical constraints, gender norms, enabling and disabling factors for adoption of innovations and government policies. These themes were generated based on our theoretical framework (more on that below). Regarding study limitations, our findings should be interpreted in context, considering the sample size and methodology used. Indeed, our overall research design is rigorous in a qualitative sense, and our findings have been validated using member-checking (Miles et al., 2014). However, we caution against the generalization of the results. Because of the purposive maximum variation sampling (Patton, 2014), selection bias is a potential limitation.

## 4 Results

### 4.1 Physical and environmental aspects of innovations

Drought and uncertainties in weather conditions were among some of the biophysical constraints to successful farming identified by both women and men, in both countries. Men and women mentioned short-term weather variations,^6^ which they have observed over the past ten years. As one young woman farmer mentioned in Morocco, “*for agriculture, the only change is the weather. Now winters are colder and dryer […] that’s why we notice a drop in production compared to 10 years ago*,” Morocco-Ain Jemaa-Youth Focus Group-Women. Indeed, some women and men mentioned specific years and months in which severe weather variations had been observed. In explaining the causes of yield loss, for example, a men farmer in Uzbekistan stressed that “*2008 was the low water year, we could not provide enough water, and respectively we lost the yield*,” UZ-Mugrak-Innovator Interview-Man. Aside from short-term weather variability, other men and women farmers mentioned long-term climatic changes that make agriculture difficult and risky. For example, in Uzbekistan, one men farmer emphasized that “*this area is quite harsh in climate and water availability; therefore, agricultural business is tough business*,” Uzbekistan-Yortepa-Middle Class Focus Group-Men. Another woman farmer in Uzbekistan echoed the forgoing point by indicating that farming is becoming increasingly challenging due to “*low volume of harvest because of climate changes or lack of water*,” Uzbekistan -Shodmonov-Lower Income Focus Group-Women.

Another biophysical constraint that came out quite strongly in the interviews was limited availability of water for irrigation. In Uzbekistan, young men farmers expressed this point most profoundly be indicating, “*wheat yield had worsened due to lack of water for irrigation and non-rainy springs*,” Uzbekistan -Mugrak-Youth Focus Group-Men. The greatest majority of men and women mentioned that due to water stress, they are unable to meet the state’s requirements for wheat production. As one man farmer in a lower income focus group mentioned, “*we face problems with water supply in agriculture and usually have problems with fulfilling state plans for wheat*,” Uzbekistan -Naiman-Lower Income Focus Group-Men.

Rust and soil salinity were also identified as biophysical constraints to agriculture especially in Uzbekistan than in Morocco. For wheat-growing and mostly men farmers, the interviews revealed that rust-related diseases had become common. In the words of one man farmer, “*rust disease is also a problem for wheat growing farmers*,” Uzbekistan -Naiman- Lower Income Focus Group-Men. Similarly, another man farmer talked about the widespread nature of soil salinity-related challenges in Uzbekistan, by saying that “*all family faces similar problems; no one has special privileges: soil salinity and lack of water are problems for everyone,*” Uzbekistan -Mugrak-Middle Income Focus Group-Men. Table 3 provides a summary of these biophysical constraints.

**Table 3:** Mentions of biophysical factors limiting production.

Biophysical constrains	Morocco (Number of mentions)	Uzbekistan (Number of mentions)
Drought and uncertainty in weather	8 times: Women (4), Men (4)	17 times: Women (4), Men (13)
Limited availability of irrigation water	8 times: Women (4), Men (4)	17 times: Women (4), Men (13)
Rust	0	3 times: Women (1), Men (2)
Salinity	0	3 times: Women (1), Men (2)
Total	16 times: Women (8), Men (8)	40 times: Women (10), Men (30)

In order to address these biophysical constraints, men farmers mentioned the adoption of two main strategies. The first was the use of new wheat varieties. These new wheat varieties were preferred by the government, who provided them to wheat growers, and by many of the farmers especially men farmers due to their ability to withstand droughts, as well as crop pests and diseases. For example, one man farmer shared that *“I started to grow new varieties of wheat such as “Krasnodar” and “Tania”. These varieties have been offered to us by the “Grain Program” as they are more productive and disease-resistant new varieties,”* Uzbekistan -Shodmonv-Farmer Innovator-Man*.* Another man farmer added, “*in our area with lack of water resources it is important to use new wheat varieties tailored to provide high yield in our conditions*,” Uzbekistan -Yortepa-Middle Class Focus Group-Men. Yield increases were also mentioned as a major reason for men farmers’ preference for new wheat varieties. A man farmer expressed this by saying, “*I have used new varieties of wheat (“Krasnodar 99” and “Tania”) and due to that I received more yield: before the yield was 4*–*4.2 ton/ha, and now my yield is 6*–*6.5 ton/ha,”* Uzbekistan -Mugrak- Farmer Innovator-Man. Despite the identified benefits of new wheat varieties, some women farmers, on the other hand, still preferred local varieties, as shown in the following interview narrative: *“I have tried to grow local wheat varieties. Because they are more fit for our local weather conditions*,” Uzbekistan -Naiman- Farmer Innovator-Woman. These preferences can be due to women’s limited access to information on improved wheat varieties but also due to their specific experiences and preferences for cultivating wheat.

Finally, crop rotation in Morocco was mentioned as another strategy by men for adapting wheat farming to recurrent biophysical changes. This strategy was mentioned more specifically to the wheat variety called *“Karim*”, which although introduced by the government about three decades ago remains one of the most cultivated varieties by farmers in Morocco (Bishaw et al., 2019). As a man farmer shared in an interview, *“rotation is important to have high yield, and Karim requires a maintained rotation to decrease the disease infection risks and preserve the land fertility,”* Morocco-Ain Jemaa- Farmer Innovator-Man. Women, on the other hand, did not mention agronomic practices in Morocco, potentially due to their limited interactions with research and extension (more on that below). We now move to discuss micro-level processes that influence the adoption of innovations before shifting attention to macro-scale policy-related factors.

### 4.2 Gender norms, household decision-making, and the adoption of innovations

Gender norms contribute significantly to constituting the social that which ultimately set a guideline for appropriate behavior for men and women in their society (Pearse and Connell, 2016, Petesch et al., 2018, Badstue et al., 2020). The adoption of agricultural innovations is highly influenced by these behaviors. The findings from both Morocco and Uzbekistan show that appropriate behavior of women is still associated with their reproductive and submissive roles while men’s behavior is under the authoritative and productive roles (See Table 4). For example, one of the lower income women farmers from Uzbekistan shared that “*a good wife/woman have to have “40 lives”. There is an Uzbek proverb – “a woman has 40 souls, lives”, that means s woman needs to be very strong; even in case she was ill, she will make her house duties as usual. She should be patient, wise, and intelligent*,” Uzbekistan -Shodmonov-lower Income Focus Group-Women. Overall, our study in both countries revealed that good women should be responsible for taking care of their family and support their husband’s decisions. Good men are responsible for being breadwinners and decision-makers of their families.

**Table 4:** Mentions of gender norms.

Mentions of gender norms	Morocco (Number of mentions)	Uzbekistan (Number of mentions)
Reproductive and submissive roles of women (idealized norm of seclusion; obedience to husband, parents, or in-laws; efficiency in completing chores and taking care of children, husbands, elderly parents and in-laws)	33 times: Women (17), Men (16)	29 times: Women (18), Men (11)
Shame around women’s work in the agricultural wage sector	4 times: Women (3), Men (1)	0
Authoritative and productive roles of men (generous, hardworking, makes enough money to attend to his family needs, take lead in decisions, takes key decisions, head of the family, fix problems)	13 times: Women (9), Men (4)	31 times: Women (15), Men (16)
Men who are good farmers seek outside advice (neighbors or extension agents)	2 times: Women (1), Men (1)	4 times: Women (3), Men (1)
Norms breaking down for women work outside the home (women as entrepreneurs; women becoming farm managers; out of necessity women must work in agriculture, out poverty or breakdown of household)	8 time: Women (3), Men (5)	9 times: Women (9), Men (0)
Women need permission and kin support for adoption of innovations (including provision of approval, information, and labour)	10 time: Women (8), Men (2)	7 times: Women (7), Men (0)
Men do not seek support of women if at all they seek support from other male kin for adopting innovations (seldom do they consult their wives, wives do not have the knowledge, wheat is a men’s domain)	13 times: Women (10), Men (3)	4 times: Women (2), Men (2)
Total	83 times: Women (51), Men (32)	84 times: Women (54), Men (30)

These norms around roles of women as supporters and men as main decision-makers and breadwinners were consistently similar across sites in the two countries and religion was enacted as justification: *“Men are in charge of women by [right of] what Allah has given one over the other and what they spend [for maintenance] from their wealth,”* Morocco-Ain Jemaa- Lower Income Focus Group-Women. Women in Uzbekistan similarly noted that *“we are Muslims, and we know that husbands are in higher position in making decisions than wives,”* Uzbekistan -Mugrak-Lower Income Focus Group-Women. The youth continued to reproduce these beliefs along with enacting religion: *“Arrijal qawamoon al nnisaa [quoting the Quran]; the man has the obligation and privilege to pay expenses and that is what makes him a man. Otherwise, if the man is there to get money from the woman, he is not “a man”,”* Morocco-Ain Jemaa-Youth Focus Group-Men. This indicates the continuation of these norms. This finding is similar to other gender norms studies within the GENNOVATE project (Badstue et al., 2020). However, these norms broke down in cases of widowhood, poverty, and outmigration of men as we will see below (See Table 4).

These norms limited women’s abilities to manage farmland in both countries. In Uzbekistan most of the women managing farmland got the land through male kin and were better off compared to women who were engaged in wheat processing. For the most part, lower-class women benefitted from the introduction of highly productive wheat varieties, which cheapened flour prices and increased its abundance, by processing them into different by-products for sale in difficult weather conditions. In Morocco, we were unable to find women involved in managing agricultural land, but women were, like in Uzbekistan, more likely to be involved in processing of wheat crop (including in paid wage work).

According to Najjar et al. (2022) during farm consolidation, the restructuring phase at which data was collected, private farmlands were drastically reduced and consolidated into larger areas averaging 33–90 ha, a pattern similar to pre-independence times. Wheat in Uzbekistan is largely a commercial crop. Although women’s role in farming is increasing as frequently reported in the sample communities and by national statistics, their abilities to cultivate as independent farmers commercially has been limited. This is due to entrenched discrimination in the *Mahalla* (where people apply for land) and in the community. Very few middle-class women benefitted from the farm consolidation policy, particularly when there were no men in the household and had to protect family land from being taken away by the state. Because male labour was not available in most of these few cases, often due to prevalent outmigration of men, this has led to land loss plunging some into poverty, and, at the very least, led to increased workloads while simultaneously opening opportunities for these women to transgress social norms related to women’s roles as farmers in their communities. Najjar et al. (2022) argue that the adoption of wheat-related innovations in Uzbekistan has reproduced and furthered gender and social inequalities with women mostly able to benefit in marginal ways. In both countries, women (of both middle and lower income) talked about the baking and processing quality of improved wheat varieties with preference for whiter colour, better taste and elasticity. In Uzbekistan, lower income women who found an opportunity to make and sell wheat by-products mixed imported Russian flour with ‘better’ local improved varieties to bring down the cost of production. Many field workers in Uzbekistan also reported that because men were paid in wheat, their wives had a readily available resource to diversify income by selling baked goods as petty traders along the roadside or in the market. Women of lower-income class confirmed this and acknowledged that newly opened private mills and bakeries created many job opportunities for them. In addition to outmigration of men, these norms tend to also break under changing economic conditions.

We found that gender norms and economic dynamism in Morocco are related in the three communities which we have selected for the study in Saiss of Ain Jemaa, Betit and Sidi Slimane. Ain Jemaa has limited natural resources endowment (rainfed agricultural conditions), and relative to the other two case studies, women were limited in their mobility. For example, they are not allowed to work outside or far away from their houses. These rigid gender norms can be also attributed to limited job opportunities (low economic dynamism) as mostly men carry out paid work in the community. By comparing across the three case studies, our findings indicate that the higher the economic dynamism, the more progressive are the gender norms, such as mobility and women’s participation in agricultural wage work. This comparative approach contributes to our understanding of how intersectionality shapes capacities to benefit from agricultural innovations based on agroecological zones and respective economic dynamism. In Betit and Sidi Slimane, women predominantly participated in the wage sector due to abundant availability of wage work opportunities year-round. In Morocco, when all agricultural tasks required to produce a single crop are considered, wheat had the largest crop-specific wage gap between men and women, at a 50% pay gap. Our interviews with wage workers in Morocco revealed that women were almost exclusively hired for sieving wheat while men were hired for a range of different activities including more permanent jobs, such as irrigation and guarding.

In group discussions, attributes of good men and women farmers were also discussed as this will affect the innovation adoption in agriculture. The expected attributes of good men and women farmers in both study countries were almost similar. Overall, it indicated that wheat is a masculine crop in both study countries, where men decide most of the marketing and decision-making part, while women farmers contribute mostly to labor (especially processing). However, it still shows that women farmers are subordinate to men farmers and support them in farming. This is evident from the comments from one lower income woman farmer in Morocco, who said “*the good woman farmer must have a “kabran” - a good trustworthy man with the sense of responsibility who looks after the land and agriculture and chooses and supervises laborers*,” Morocco- Ain Jemaa-Lower Income Focus Group-Women.

**Fig. 2:**
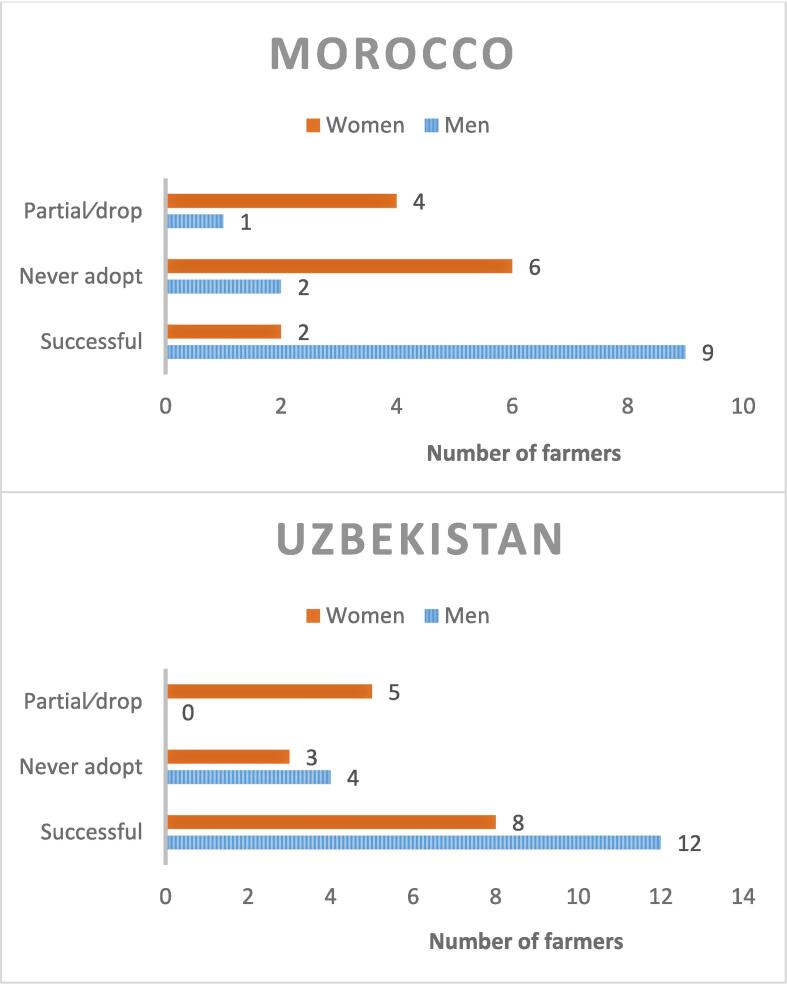
Innovation adoption status in Morocco (n = 24) and Uzbekistan (n = 32) * Source: Innovator and life history interviews.

The study results showed that men adopt new innovations faster compared to women farmers (Fig. 2). The data further revealed that women farmers in Uzbekistan were more successful in adopting innovations compared to Morocco. In general, women seemed to be willing to copy rather than initiate the adoption of an innovation in study area. For example, one poor woman farmer in Morocco shared that “*whatever the neighbors do, we do it. We are not scared to try out new things,*” Morocco-Ain Jemaa- Lower Income Focus Group-Women. Some women felt that improved wheat varieties are the domain of their husbands and does not fall under their responsibilities. This perception was more evident in the stories of lower income women from Morocco: *“Women never interfere with their husbands’ decisions about growing improved wheat varieties. For selected seeds, husbands consult with sellers, the association, and/or people in agricultural guidance / advisory*,” Morocco-Ain Jemaa- Lower Income Focus Group-Women.

Along the same lines, findings from Morocco reveal that women felt it was not their domain to learn about new wheat varieties: “*They did an experiment on a variety of wheat called “Rihan” and chickpeas last year and this year, and we do not know… it is the men who know these thing*s,” Morocco-Ain Jemaa- Middle Income Focus Group-Women. Middle class women, nonetheless, felt that they have a role in adoption of wheat varieties that of testing its baking quality: “*Women baked new varieties of wheat (Rihan) and tell their husbands about the quality of bread. However, they do not decide which variety to cultivate*,” Morocco-Ain Jemaa- Middle Income Focus Group-Women. Their role, as they have explained it, is to give suggestions rather than to make varietal adoption decisions. Women from lower class reported that their involvement depends on the husband’s approach for decision-making in adopting improved varieties: “*Some men consult with their wives or children, and sometimes they do what they want*,” Morocco-Ain Jemaa- Lower Income Focus Group-Women. When asked whether they need to consult with someone to adopt improved varieties, some women from the lower income group on the other hand said: “*Women of course consult with the men in almost everything and anything*,” Morocco-Ain Jemaa- Lower Income Focus Group-Women. These findings were consistent across class and both study countries. *“We live in a Muslim society: if a husband does not support his wife, she will fail,”* Uzbekistan-Mugrak- Middle Income Focus Group-Women. Overall, this study showed that women needed support of husbands and kin alike to adopt and/or adapt new wheat varieties (See Table 4). This is directly linked to their decision-making capacity at the household level.

Moreover, the study findings revealed that for young women permission and support from husband and mother in-laws was needed for women to decide on innovation adoption. This hindered the women from attempting to innovate in the first place. Similarly, Bossenbroek et al. (2015: 348) note that in Saiss region for young women to fulfill their dreams would require changing *“patriarchal family and kinship hierarchies and challenging the gender ideologies that help keep these in place”.* Young men, albeit having more decision-making power than young women, also felt disadvantaged in their ability to adopt innovations and benefit from them, as one young man in Morocco explains, *“Young people could make decisions about adopting agricultural innovations, but they have no financial means,”* Morocco-Betit-Youth Focus Group-Men.

However, as women advance in age, they usually gain more freedom, as illustrated in the following quote: *“I am not a young woman anymore. I think only about my children, and nobody would look at me as a woman. No man would be attracted to me anymore; this gives me a little bit of freedom to go out. My husband will not prohibit me like before,”* Morocco-Ain Jemaa-Life History Interview-Woman. In addition to age easing norms, women of lower income were less likely able to follow social norms. Our interviews with wage workers revealed, nonetheless, that shame and tension with husbands for working in agriculture were commonplace in these households. The introduction of irrigation in Morocco, in particular, created job opportunities mainly for women especially in irrigated cash crops, such as fruits, potatoes and onions. Due to reputational damage, because of having to stand near unrelated men and the high incidences of sexual harassment, many women who are wage workers reported being ashamed of their engagement in the wage sector.

Many resorted to hiding their faces with only their eyes showing and leaving at the crack of dawn in fear of getting exposed. This was particularly a problem for the young and unmarried wage workers. Some worked around these norms by working for farmers that their husbands knew or rarely for women farmers. The following quote shows the shame that men in the community held should their wives work in the wage sector, *“husbands do not accept that their wives work for a male farmers/supervisor. I prefer starving over my wife working for other men,”* Morocco-Ain Jemaa-Lower Income Focus Group-Men. Yet, even women who worked on locally known farmers’ lands still experienced reputational damage due to deeply entrenched norms of seclusion which put tension on their relationships with their husbands: *“I used to work for women farmers. I never worked from the mawqaf [the place were men and women labourers aggregate to find wage work on commercial or private farms]. Women who visit the mawqaf and are known to the community experience significant reputational damage… Yet others started to say to my husband that he was giving me too much liberty and our relationship got negatively affected and changed. He no longer allows me to work,”* Morocco-Sidi Slimane-Life History Interview-Woman.

We found that some women called all wheat varieties using the generic term of *“technique”* (meaning non-local varieties). This was largely due to their exclusion from extension advice which is often tied to the owner of the land. This was evident by a powerful quote from a man farmer in Morocco, *“women have no relation with agricultural extension agents because they talk only with men,”* Morocco-Ain Jemaa-Middle Income Focus Group-Men. However, even in such patriarchal context in Morocco, husbands and in-laws provided some support to women farmers in innovating especially when they had fulfilled the good wife roles. In Uzbekistan, 14 women farmers who have adopted wheat innovations reported that lack of husband and in-laws support led to dis-adoption. They further reported that if permission was granted, it could also lead to workload reduction and more equitable division of labor by family members (husbands, sons, in-laws). Mothers-in-law in Uzbekistan control daughters-in-law’s decisions in households, competing over power in the household, as described by a men farmer in Uzbekistan *“when housewife and mother-in-law live in one house, there are tensions between the two in understanding and value of family relations, children upbringing, house tidiness and duties,”* Uzbekistan-Mugrak-Middle Income Focus Group-Men. With the outmigration of men, this relationship is changing to supportive when their livelihoods become dependent on daughters-in-law. For example, a married woman from Shodmonov shared her story that *“my mother-in-law now fully supports and permits me to do the business of selling bread. She registered the house in my name to show her support because she was angry at her son that he married in Russia without her permission, and her son’s action was shameful for her. She felt guilty toward me and my children”.*

While all the aforementioned factors we found to affect innovations, macro-level policy-related factors were also at play, as shown in the next section.

### 4.3 Policy issues affecting the capacity to innovate

This part of the results section presents findings linked to existing policies enabling or inhibiting farmers’ capacities to innovate. Overall, the study showed that men were far more likely to report government policies as either hindering or enabling their innovation capacities in both countries (see Table 5). The major policies affecting farmers in both study countries is land privatization. This policy in Morocco has led to credit opportunities among both men and women farmers at the local level whereby land ownership and subsequent membership in cooperatives was a necessary precondition for access to credit, in particular for digging wells, but this was accompanied with reduced subsidization and investment in agriculture, thus affecting farmers’ access to improved seed varieties. Bishaw et al. (2019: 89) find that “among the top 10 varieties, four, which are all at least 24 years old, cover 56% of the total wheat area – showing that old varieties still dominate the Moroccan wheat fields”. Their survey findings reveal that farmers are not aware of the newer improved varieties released by the government, and when they are, the seeds are not available for adoption. The majority of women and younger men neither owned land nor consequently joined farmers’ cooperatives. The GMP increased the investment in drip irrigation in Morocco, which was considered good progress by men farmers. Wells were considered good economic investment and loans for wells were provided largely to men farmers through cooperatives (Faysse, 2015). While married women reported being more confined to their households due to higher reliance on hired wage work which came with cash crop cultivation enabled by irrigation wells (findings also reported in Saiss by Bossenbroek and Zwarteveen, 2015), women in the poor and landless households benefited from wage opportunities but had to content low pay as well as precarious and seasonal working conditions.

**Table 5:** Mentions of government policies.

Mentions of government policies	Morocco (Number of mentions)	Uzbekistan (Number of mentions)
Subsidies for drip irrigation	2 times: Women (0), Men (2)	0
Credit provision	2 times: Women (1), Men (1)	8 times: Women (3), Men (5)
Other support for farmers (extension advice, machinery, infrastructure, marketing)	4 times: Women (2), Men (2)	5 times: Women (2), Men (3)
Distribution of wheat seeds	0	6 times: Women (0), Men (6)
Land privatization	1time: Women (0), Men (1)	3 times: Women (2), Men (1)
Critical mentions of government policies (feelings of abandonment, lack of enough seeds, delayed and slow services)	1 time: Women (0), Men (1)	3 times: Women (0), Men (3)
Total	10 times: Women (3), Men (7)	24 times: Women (7), Men (18)

However, class and the need to have land and money to co-subsidize also affected investment in drip irrigation. The land privatization policy which stipulated that only one person, often a man, can own the land marginalized other men and especially women family members and fueled family conflict, including violence, and landlessness. One men key informant told us that a man in the community stabbed his sibling for lack of reaching an agreement over who gets the land title. Another woman reported that her husband was kicked out of the extensive family home and farm by his brother who owned the land and that this has made them poorer. She now works in the agricultural wage sector. Monocropping and a focus on improved crops was found to be increasing, but this also increased gender and class inequalities albeit creating meagre wages for lower class women (Kandiyoti, 2003, Bossenbroek and Zwarteveen, 2015). In Uzbekistan, the re-consolidation of land to achieve efficiency gains were found to be helpful in the expansion of land area for those who are able to fulfill the state’s quotas. However, this forced mostly men farmers to lose the land and many have migrated to cities and Russia creating opportunities for a few women to manage land (Djanibekov et al., 2012).

One men farmer indicated that “*ten years ago, micro-credits were not available and access to “Credit Agricole” is difficult, due to the absence of land titles. Even though we get the loan, the loans of “Credit Agricole” are hard to reimburse due to regular drought and farmers have to lose their lands*,” Morocco-Ain Jemaa- Lower Income Focus Group-Men. This concern was representative of accounts given by most other farmers in both study countries, which provides evidence that neoliberalism is a double-edged sword, which is helpful (by providing mostly landowners with a loan) but at the same time it also inhibits (by taking land from farmers). One of the poor women farmers from Morocco mentioned, “*men farmers were the major recipients of the loans. Because, for a woman farmer, it’s more difficult to apply to any state bodies or financial institutes because of negative attitudes from men towards women,*” Morocco-Betit- Lower Income Focus Group-Women. Our findings reveal that men who were able to access these lands installed wells on their land and intensified their agricultural production and expanded their areas cultivated. Very few women mentioned taking formal loans and they were of smaller value. According to Faysse (2015) loans in the GMP, which has a substantial budget for strengthening agricultural contributions to the national economy, are mostly tied to landowners.

The land privatization policy in Uzbekistan was that the government provided expansive credit to landholders and was indifferent to their gender with a sustained focus on reaching self-sufficiency in wheat production. Similarly, distribution of home plots in Uzbekistan helps women as cashier of the household striving to achieve self-sufficiency; however, limited availability of land is the major drawback. Not all households were able to access home plots. The credit and privatization policy further focused on wheat and cotton crops in Uzbekistan, which according to the farmers, was helpful in increasing wheat yields. “*But state credits are provided only for cotton and wheat production and for machinery purchase. For other purposes we can hardly get credits or loans from the banks as they require pledge, and we have nothing valuable to pledge*,” Uzbekistan-Yortepa-Life History Interview-Man. Many women and men farmers explained that they also preferred that the state focused its extension advice and provision of credit and inputs to cultivating fruits and vegetables for earning a higher income, boosting household consumption, and diversifying income. The sustained focus of the state on wheat and cotton, however, provided limited profits for farmers, while also limiting water and land and other resources available for other crops and livestock enterprises (Djanibekov et al., 2012, Rudenko et al., 2012)..

Most of the farmers also reported lack of seed availability, leading to delays in adoption of improved varieties. However, the agrarian reform in Uzbekistan offered several women-specific projects by providing funding to women enterprises. But most of these women were confined to traditional domains of sewing and baking rather than adopting new technologies and innovations in farming. Our findings support prior assertions in post-Soviet Uzbekistan of the government being interested in perpetuating traditional roles for women, in what has been termed by the state as a return to ‘lost traditions’ (Kandiyoti, 2007, Lombardozzi, 2022). This was evident from the stories of one of the women farmers: *“as for me I learned about flour a little bit as I wanted to teach girls to bake. I tried much flour that was made from local wheat varieties as well as from foreign varieties. And I came to one opinion that local ones are good for traditional foods but not for cakes: I mean for confectionery, you need the other foreign one*,” Uzbekistan-Mugrak-Farmer Innovator Interview-Woman. Similar findings are reported for the MENA region whereby agricultural programs and support for rural women is often in traditional and domestic domains (Najjar et al., 2019, Ragetlie et al., 2021). In their study on women’s involvement in irrigation in Egypt, Najjar et al. (2019) found that women were often trained on domestic water use and were largely excluded from irrigation training and innovations despite their significant involvement in irrigation activities which was deemed a masculine domain by local people and government officials alike.

## 5 Discussion and conclusion

Using a feminist political ecology analytical framework, this study sought to answer two main questions: How does gender (across generations, locations, class and marital status) affect agricultural technology adoption, specifically wheat innovations? What is the role of biophysical constraints and broader macro-economic factors in the adoption of wheat innovations? As demonstrated in the study findings, the complex interactions of biophysical constraints, intrahousehold relations, and the broader macro-level political economy of agriculture converge to influence farmers’ wheat production practices. Men were enabled to choose new wheat varieties which was shaped not only by climate variability, rust-related diseases, and soil salinity, but also the everyday decision-making processes at the household level. In Uzbekistan with the outmigration of men, mostly due to difficult and unprofitable farming conditions, women are gaining more freedom to adopt new agricultural innovations (farming land for middle class women and sale of wheat by-products for lower class women) and gaining support from the government who wants to meet its goal of self-sufficiency in wheat production and whose kin survival becomes dependent on them (Najjar et al., 2022). Overall, our findings showed that there was a strong notion of women as housekeepers and men as breadwinners in both countries. Due to this notion, women are not taken seriously as farmers, so they gained limited roles and power in public spheres (e.g., in cooperatives and Water User Associations (WUAs) in Uzbekistan). Women were found to be getting limited advice from agricultural extension agents, even those working on their husband’s lands. These findings are similar to other case studies from South Asia, such as in India, Nepal and Bangladesh (Agarwal, 2001, Devkota et al., 2016, Gartaula et al., 2020). In both Morocco and Uzbekistan, the majority of women were implicated in wheat processing (whether for sale or for household consumption). Some women exercised their agency to influence their husbands’ adoption decisions to more suitable varieties while others increased profits from sale of wheat by-products.

Moreover, while biophysical constraints, like the limited availability of water for irrigation equally affect both men’s and women’s wheat production practices, men were found to adopt wheat innovations much faster than women. This higher adoption rate is influenced most crucially by men’s dominated roles in agricultural decision-making. In turn, this role, along with the increase in decision-making power that it entailed, involved evoking religion as justification in everyday life by women and men alike and across generations. This contract, however, was broken in cases of widowhood, outmigration of men, economic changes, and lack of interest on the part of men. A growing body of FPE case studies has documented similar findings indicating that men’s increasing decision-making power is a factor that enhances their innovation capacities (e.g., Bassett, 2002; Bezner Kerr, 2014), thus demonstrating the need to take intra-household relations seriously in the transformation of farming systems under changing ecological conditions. In terms of geographical coverage of FPE empirical cases, less has been written on Morocco and Uzbekistan. Gender inequality persists in these regions because of an entrenched patriarchy from within the private domain of households, to communities, to a more systemic patriarchy sustained by the state and other key political and economic institutions.

Thus, the findings here add to our understanding of gender, intersectionality, and natural resources management in less-studied countries. Until more recently, the first generation of FPE research (e.g., Bassett, 2002; Bezner Kerr, 2014, Carney, 1993) had little to say about intersectionality. Our case study is therefore a modest contribution to the growing literature on intersectional FPE. Women’s inability to adopt new wheat varieties at a rate as fast as men is also explained by the broader macro-level political economy of land access. In both case study countries, land privatization is an issue that came out quite strongly as a barrier to the adoption of wheat innovations and subsequent validation of women as farmers in their own right. As the FPE literature on agriculture has persistently shown (e.g., Baada et al., 2019; Bezner Kerr, 2014, Hovorka, 2006), in order to understand farm management constraints, there is a need to move beyond intra-household politics or gender norms and also examine political-economic policies over which farmers have little control and the role of kinship, class, social and marital status. In the particular study examined here in both Morocco and Uzbekistan, governments’ policies on land were found to be reworking farming systems in complex ways, with negative implications for wheat innovations (Kandiyoti, 2003, Najjar et al., 2022, Bossenbroek and Zwarteveen, 2015).

This led to a situation whereby most of the benefits that accrued to women were related to wage work, mostly for poor women (married or heads of households), and processing of wheat by-products, including for profit. Few women from the middle class in Uzbekistan who had no husbands or uninterested husbands managed to benefit from wheat innovations at the production phase where most of the benefits accrued in terms of social status and monetary gains. This was not the case in Morocco. Aside from land privatization, women also mentioned lack of access to agricultural extension experts who have crucial information on the adoption of wheat innovations. This stems from the erroneous notion that women are not farmers, even in cases where they provide significant labor on their husband’s fields. Another reason is women’s subordinated position (see similar reported research findings for Syria and Egypt, respectively, Galiè et al., 2013, Najjar et al., 2019). To this end, gender remains an integral part of and key element to understanding women’s inability to scale up wheat innovations even when resources are largely available. Most of the gender research focuses on spousal relations; in this paper we have shown the role that mothers-in-law and the life cycle have in gaining control over decision-making in agricultural innovations.

The analysis provided here offers several important implications for crafting gender-sensitive policies to promote wheat innovations more specifically, and smallholder farming systems more broadly. Addressing the combined effects of environmental change, household-level gender politics, and government policies on agriculture is crucial for addressing and lessening gender-based differences in agricultural innovations. Such an approach can foster social resilience^7^ in the light of growing climate variability, among some of the biophysical constrains mentioned by mostly men farmers in this study. Moreover, more than providing new crop varieties—in this case wheat–is needed in any attempt to address the current and specific challenges confronting different types of farmers in Morocco, Uzbekistan, and beyond. The diffusion of such seeds should be accompanied with social interventions, such as workshops aimed at discussing cultural and social issues that affect agriculture. The government of Morocco and Uzbekistan are well advised to integrate social, economic and agricultural productivity outcomes into their agricultural programming (Faysse, 2015, Bossenbroek and Zwarteveen, 2015, Najjar et al., 2022). Our study demonstrated that most of the credit programs are biased to men landowners which have contributed to more profitable farming, maintaining land titles, and accessing irrigation for these middle class men; while landless men and especially women benefitted through low paid and seasonal wage work opportunities. We found very few microcredit programs for women, and they were of comparatively smaller values. Without focusing on the socio-cultural factors affecting agriculture, new seed varieties alone in themselves cannot address the multifaceted problems confronting men and especially women farmers in all parts of the world. Along the same lines, and particularly for Uzbekistan, broadening the government’s focus to other crops and livestock enterprises beyond wheat stands to build the resilience, respond to the needs as well as improve income and nutrition of men and women farmers (Djanibekov et al., 2012, Rudenko et al., 2012, Najjar et al., 2022).

## Declaration of Competing Interest

The authors declare that they have no known competing financial interests or personal relationships that could have appeared to influence the work reported in this paper.

## Data Availability

The data that has been used is confidential.

## Footnotes

5 The first innovation, conservation agriculture, also known as ‘no-tillage’ and ‘direct planting’, was introduced by the Institut National de la Recherche Agronomique (INRA) and the International Center for Agricultural Research in the Dry Areas (ICARDA) on a few farmers’ fields to showcase the approach and its benefits to the farmers.The second innovation is the diversification of wheat-based systems for enriching soil fertility by introducing food legume crop rotations and improved phosphorus fertilizer management. This activity focused on cultivating and fertilizing winter chickpeas to increase yield and use market niches.The third innovation also involved cereal and legume systems and is concerned with promoting already proven Integrated Pest Management (IPM) options for increasing crop productivity at the farm level and developing new IMP options for managing emerging biotic constraints for the cereal and food legume systems.

6 Short-term is within the living memory while long-term is what was not witnessed first-hand and heard about from others.

7 Social resilience is a concept that entails the ability to cope with, tolerate or adjust to environmental and social threats of various kinds (Keck and Sakdapolrak, 2013).
